# Secondary Syphilis in Cali, Colombia: New Concepts in Disease Pathogenesis

**DOI:** 10.1371/journal.pntd.0000690

**Published:** 2010-05-18

**Authors:** Adriana R. Cruz, Allan Pillay, Ana V. Zuluaga, Lady G. Ramirez, Jorge E. Duque, Gloria E. Aristizabal, Mary D. Fiel-Gan, Roberto Jaramillo, Rodolfo Trujillo, Carlos Valencia, Linda Jagodzinski, David L. Cox, Justin D. Radolf, Juan C. Salazar

**Affiliations:** 1 Centro Internacional de Entrenamiento e Investigaciones Médicas (CIDEIM), Cali, Colombia; 2 Centers for Disease Control and Prevention, Atlanta, Georgia, United States of America; 3 Secretaria de Salud Publica Municipal, Cali, Colombia; 4 Department of Pathology, Hartford Hospital, Hartford, Connecticut, United States of America; 5 Fundación Clínica Valle del Lili, Cali, Colombia; 6 Walter Reed Army Institute of Research, Rockville, Maryland, United States of America; 7 Department of Medicine, University of Connecticut Health Center, Farmington, Connecticut, United States of America; 8 Department of Genetics and Developmental Biology, University of Connecticut Health Center, Farmington, Connecticut, United States of America; 9 Department of Pediatrics, University of Connecticut Health Center, Farmington, Connecticut, United States of America; 10 Division of Infectious Diseases, Connecticut Children's Medical Center, Hartford, Connecticut, United States of America; University of Washington, United States of America

## Abstract

Venereal syphilis is a multi-stage, sexually transmitted disease caused by the spirochetal bacterium *Treponema pallidum (Tp)*. Herein we describe a cohort of 57 patients (age 18–68 years) with secondary syphilis (SS) identified through a network of public sector primary health care providers in Cali, Colombia. To be eligible for participation, study subjects were required to have cutaneous lesions consistent with SS, a reactive Rapid Plasma Reagin test (RPR-titer ≥1∶4), and a confirmatory treponemal test (Fluorescent Treponemal Antibody Absorption test- FTA-ABS). Most subjects enrolled were women (64.9%), predominantly Afro-Colombian (38.6%) or mestizo (56.1%), and all were of low socio-economic status. Three (5.3%) subjects were newly diagnosed with HIV infection at study entry. The duration of signs and symptoms in most patients (53.6%) was less than 30 days; however, some patients reported being symptomatic for several months (range 5–240 days). The typical palmar and plantar exanthem of SS was the most common dermal manifestation (63%), followed by diffuse hypo- or hyperpigmented macules and papules on the trunk, abdomen and extremities. Three patients had patchy alopecia. Whole blood (WB) samples and punch biopsy material from a subset of SS patients were assayed for the presence of *Tp* DNA polymerase I gene (*pol*A) target by real-time qualitative and quantitative PCR methods. Twelve (46%) of the 26 WB samples studied had quantifiable *Tp* DNA (ranging between 194.9 and 1954.2 *Tp pol*A copies/ml blood) and seven (64%) were positive when WB DNA was extracted within 24 hours of collection. *Tp* DNA was also present in 8/12 (66%) skin biopsies available for testing. Strain typing analysis was attempted in all skin and WB samples with detectable *Tp* DNA. Using *arp* repeat size analysis and *tpr* RFLP patterns four different strain types were identified (14d, 16d, 13d and 22a). None of the WB samples had sufficient DNA for typing. The clinical and microbiologic observations presented herein, together with recent Cali syphilis seroprevalence data, provide additional evidence that venereal syphilis is highly endemic in this region of Colombia, thus underscoring the need for health care providers in the region to be acutely aware of the clinical manifestations of SS. This study also provides, for the first time, quantitative evidence that a significant proportion of untreated SS patients have substantial numbers of circulating spirochetes. How *Tp* is able to persist in the blood and skin of SS patients, despite the known presence of circulating treponemal opsonizing antibodies and the robust pro-inflammatory cellular immune responses characteristic of this stage of the disease, is not fully understood and requires further study.

## Author Summary

Venereal syphilis is a sexually transmitted disease caused by the bacterium *Treponema pallidum* (*Tp*). We describe 57 patients (age 18–68 years) from Cali, Colombia diagnosed with secondary syphilis (SS). Most were women (64.9%); predominantly Afro-Colombian (38.6%) or mestizo (56.1%), and all of low socio-economic status. Three (5.3%) were newly diagnosed with HIV infection at study entry. The typical palmar and plantar rash of SS was the common clinical finding (63%). Whole blood (WB) samples and skin biopsies were assayed for *Tp* DNA by using molecular methods. 46% of the WB samples had circulating *Tp* DNA and 64% were positive when the DNA was extracted on the same day of collection. *Tp* DNA was also present in the skin of 66% (12/26) of biopsies tested by PCR. We conclude that primary care providers in countries like Colombia need to remain highly vigilant for the clinical presentation of SS. The study also provides, for the first time, qualitative and quantitative evidence that untreated SS patients have significant numbers of spirochetes in blood and skin, and that this occurs despite the known presence of circulating anti-treponemal antibodies and strong cellular immune responses associated with this stage of the disease.

## Introduction

Syphilis is a sexually transmitted disease (STD) caused by the spirochetal bacterium *Treponema pallidum (Tp)* subspecies *pallidum* [1], [2]. Despite the existence of inexpensive and effective antibiotic treatment regimens, syphilis continues to be a major public health problem. According to the most recent World Health Organization (WHO) estimates, approximately 10.6 million new syphilis cases occur yearly throughout the globe [3]. Although venereal syphilis has re-emerged in developed countries [4], most individuals (>90%) who acquire the disease reside in less affluent regions of the world [3]. In Cali, Colombia, our Latin American study site, the yearly incidence of venereal syphilis over the last decade was estimated to be around 32 cases per 100,000 (GE Aristizabal, City of Cali Health Department's STD Division - personal communication). This approximation is significantly higher than in Western Europe [5] or in the United States [4], and based on the very high rates of well documented gestational and congenital syphilis rates in the city [6], it very likely underestimates the true prevalence of venereal syphilis in Cali. Indeed, in a recent study conducted by our collaborators [7], 1.2–6.8% of 23,190 sexually active 15–24 year old men and women, from different socioeconomically deprived Cali districts “comunas”, had serum RPR values ≥1∶8. In the same study, 13.8% of men who have sex with men (MSM), 28.8% of female commercial sex workers and 39.2% of transsexuals in Cali were also found to be sero-positive for syphilis [7]. Strategies to control sexual transmission of *Tp* are, thus, urgently needed in Colombia, not only because of the harmful consequences of syphilis to infected pregnant women or their unborn children [5], [8], [9], but also because of the strong association of venereal syphilis with an increased risk for acquiring and transmitting human immunodeficiency virus (HIV) [4], [10]–[12]. To begin to curb the spread of venereal syphilis it is very important that health care providers become more adept at distinguishing the typical and atypical signs and symptoms associated with early syphilitic infection.

Unlike most invasive bacterial infectious diseases, venereal syphilis is a multistage illness with clinical manifestations that reflect the propensity of *Tp* to disseminate systemically and to induce persistent chronic inflammation in diverse tissues and organ systems [1], [2], [13], [14]. Infection begins when the bacterium comes in contact with skin or mucosal membranes, multiplying locally over several days, while simultaneously disseminating through blood vessels and lymphatics [2], [15]. The appearance of a painless ulcer, more commonly known as a “chancre”, typically only appears 2–4 weeks after the initial contact with the spirochete [2], [15], [16]. By this time, organisms have disseminated from the primary site of infection to various organ systems and throughout the dermis [2], [15], setting the stage for what is classically known as secondary syphilis. This stage of the disease, which is the principal focus of the current study, is characterized by the most overt systemic clinical features, including a variety of dermal manifestations as well as systemic signs and symptoms typically appearing within 4–10 weeks of the initial infection [13], [14]. Despite the evolving nature of the adaptive immune response [17], including the presence of opsonic antibodies [18], it may take weeks and in some cases months for the host to gain the upper hand against the invading pathogen, ultimately giving rise to an asymptomatic stage known as latent syphilis. During early latency (the first 4 years post-infection) patients may experience recurrences of spirochetemia as well as clinical relapses [13], [15], [19], both indicative of the host's inability to fully eradicate and control the bacterium. In due course, patients enter late latency and several years later 15–40% of them develops recrudescent forms of the disease; collectively referred to as tertiary syphilis [14], [20], [21]. How the bacterium disseminates from its primary or secondary sites of infection, and why it persists in its human host for extended periods of time despite the vigorous cellular and humoral adaptive immune responses it evokes [2], [17], remains unresolved.

Greater progress towards deciphering the pathogenesis of the paradoxical nature of venereal syphilis has been hampered due to the inability to readily propagate *Tp in* vitro, as well as the lack of a suitable inbred animal model to study the disease. Nevertheless, much can be learned about the pathogenesis of venereal syphilis through a combined analysis of the epidemiologic, clinical and microbiologic features of the various stages of the disease. In the current study we review the clinical, histopathologic and laboratory features of 57 patients diagnosed with SS through a network of public sector primary health care providers in Cali, Colombia. Concurrently we determined spirochetal DNA burdens in WB and skin samples from a subset of these patients by using a highly sensitive real-time PCR assay. In conjunction with available Cali syphilis seroprevalence data [7], this study provides clinical and microbiologic evidence that venereal syphilis is highly endemic in this region of Colombia. Our findings also make evident that in this population early syphilis patients may go undiagnosed and untreated for several weeks, in some cases for several months. We also provide, for the first time, quantitative and/or qualitative molecular evidence that spirochetes are present in significant numbers in skin and blood of untreated SS patients. Paradoxically, spirochetes persisted despite the known presence of circulating antitreponemal opsonizing antibodies in the serum of SS patients [18], as well as the robust pro-inflammatory dermal cellular immune response characteristic of this stage of the disease [17].

## Materials and Methods

### Subject Recruitment

**Figure 1:**
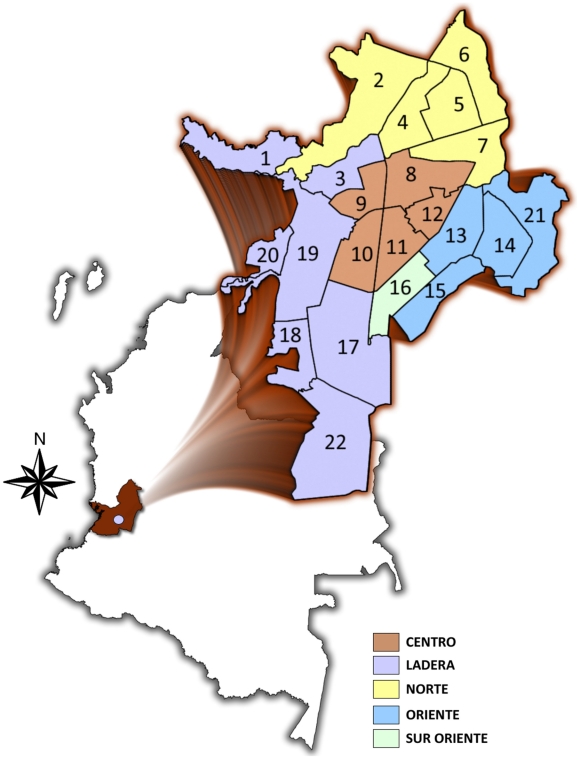
Study site and syphilis network. The city of Cali is located in south-western Colombia (see inset). Cali is divided into 22 (numbered in the figure) distinct geographic districts called “comunas”. These are clustered within 5 administrative regions called Empresas Socials del Estado “ESEs”: Ladera (yellow), North (blue), Center (pink), South-East (purple) and East (green). Public health hospitals are strategically distributed within the “ESEs”. The syphilis patient recruitment network was comprised of 13 hospitals and health centers located throughout the city.

SS patients from Cali, Colombia were recruited from 2003 to 2009 as part of an ongoing syphilis immunology study [10]. Cali is the 3^rd^ largest city in Colombia with a population of 2,139,535; many of whom belong to middle or low socio-economic strata (32.1% and 31.6% respectively). Out of 22 distinct comunas in the city, 11 are considered very poor, have less than adequate access to health care services and as already alluded to above, very high RPR seropositivity rates [7]. Five public health institutions called Empresas Sociales del Estado (ESEs) are strategically located throughout the city, and are responsible for providing regional health care to the local population residing in the various comunas (Figure 1). Prior to study initiation, nurses and physicians working in decentralized hospitals and health care centers affiliated to individual ESEs were trained by study personnel to properly recognize and treat early syphilis patients. Patients who met criteria for a diagnosis of SS and who agreed to participate in the study were referred by participating providers to the “Centro Internacional de Entrenamiento e Investigaciones Médicas” (CIDEIM) for further examination by study physicians and for confirmatory blood tests. At CIDEIM all participants were required to sign informed consent. Study procedures were reviewed and approved by the human subjects boards at the Connecticut Children's Medical Center, the University of Connecticut Health Center (UCHC), Walter Reed Army Institute of Research (WRAIR) and CIDEIM. The study protocol was also reviewed by ethics committee from each of the participating ESEs. The Institutional review board at the Centers for Disease Control and Prevention (CDC) approved the molecular analysis of *Tp* DNA in blood samples and skin biopsies obtained from secondary syphilis patients and controls.

### Case Definition and Study Procedures

The diagnosis of secondary syphilis was based on a compatible medical history, the appearance of characteristic skin or mucosal lesions (see below), a reactive RPR of ≥1∶4 dilutions, and a positive confirmatory treponemal test (Fluorescent Treponemal Antibody Absorption, FTA-ABS). All subjects had a complete physical examination performed by one of two trained dermatologists (AC or RT) who are well versed in the identification of venereal syphilis. Individual case histories, serologic data, and photographs of dermal and cutaneous lesions were then reviewed by at least one of two infectious disease experts (JR or JS). Serologic tests for syphilis and HIV were conducted for all participants at a centralized reference laboratory in Cali. Study subjects were not eligible for participation if they were <18 years old, if they were known to be HIV+ or if they had used antibiotics within 4 weeks prior to study entry. Pregnant women were also excluded from participation. Socio-demographic characteristics and relevant clinical and epidemiological information were obtained by means of a standardized questionnaire. Study dermatologists obtained skin biopsies from 11 patients who had representative syphilis dermal lesions. Whole blood and skin biopsies were also collected from a subset of patients for real time polymerase chain reaction (PCR) *Tp* DNA quantitation (see below). All patients were treated with 2.4 million units of intramuscular benzathine penicillin in accordance with Colombian public health guidelines, which are in accord with available CDC treatment guidelines [22]. Patients were asked to return within 45–60 days for a follow-up clinical examination and repeat RPR titers. When possible, partner notification and treatment were done by health care providers from the city of Cali's health department. Diagnosis and treatment of additional STDs was done through the patient's primary health care provider.

### Clinical Laboratory and Histologic Studies

All patients had non treponemal (RPR), and treponemal (FTA-ABS) tests performed at a reference laboratory in Colombia. An ECLIA (Electro-chemiluminescence Immunoassay) HIV serum antibody test, a complete blood count (CBC) with manual differential, and quantitation of the erythrocyte sedimentation rate (ESR) were also done. All women underwent a serum pregnancy test (none were positive). The three positive HIV ECLIAs identified in study patients were subsequently confirmed by Western-blot analysis. HIV-positive patients were referred to a public health care sector HIV clinic for additional testing and treatment. Dark field microscopy was not required in this study. Punch biopsies from affected dermal sites were obtained by the study dermatologist from 11 patients deemed to have distinctive clinical manifestations classically associated with SS. Available tissues from these patients were stained with both hematoxilin-eosin (H&E) and Warthin-Starry silver stain (one (9%) revealed spirochetes by silver stain). Individual biopsy samples were then systematically analyzed by trained pathologists in Colombia and subsequently corroborated by a pathologist in the United States.

### Molecular Studies

The clinical sensitivity of the *pol*A real-time PCR was determined by testing blood spiked with either purified *Tp* DNA or *Tp* organisms. Fifty milliliters of peripheral blood was collected in EDTA tubes from a single donor. The blood sample was maintained at room temperature prior to performing spiking experiments. *Tp* was grown in the testis of New Zealand white rabbits and harvested as previously described [23]. The cultivation of *T. pallidum* in rabbits was approved by the CDC animal care committee. Treponemes were diluted to a concentration of 1.5×10^7^/ml using prewarmed TpCM (*Tp* cultivation medium). A ten-fold serial dilution of the suspension was performed using prewarmed TpCM and a 200µl aliquot of each ten-fold dilution was added in duplicate to 1.8 ml of blood. One set of tubes was kept at room temperature for 1 hr while the second set was placed in a refrigerator for 26 hrs. DNA was extracted from a 200µl aliquot of the 10^−1^ dilution of *Tp* organisms in TpCM using the QIAamp DNA Mini Kit (Qiagen, Valencia, CA). The purified DNA was serially diluted ten-fold in sterile PBS and a 200µl aliquot of each ten-fold dilution was added in duplicate to 1.8 ml of blood. Tubes were left at room temperature for 1 hr or stored at 4°C as for blood spiked with *Tp* organisms. DNA extraction from all spiked blood samples was achieved using the QIAamp DNA Midi kit (Qiagen) and the purified DNA was eluted in 500µl Buffer AE. A 10µl sample was tested in duplicate using a real-time PCR that targets the DNA polymerase I gene of *Tp* (*pol*A).

Five to ten milliliters of whole blood were collected in tubes containing EDTA (1.8mg EDTA per milliliter of blood) from 26 SS patients enrolled during the last three years of the study. The first 15 WB samples collected were stored frozen for several days and subsequently shipped on dry ice overnight to UCHC. DNA was subsequently extracted from 0.4 ml of whole blood using the QIAamp DNA Blood Mini Kit (Qiagen) following procedures recommended by the manufacturer. For the last eleven patients enrolled into the study DNA was extracted from whole blood samples on site by CIDEIM personnel, and subsequently shipped on dry ice to UCHC. A 4 mm-punch skin biopsy of SS lesions was also obtained from the same group of patients and controls, snap-frozen and stored in liquid nitrogen in preparation for overnight transportation on dry ice to UCHC. Skin from three healthy controls was also obtained at CIDEIM and handled in the same fashion. Upon arrival at UCHC, DNA was extracted from all skin samples using the Qiagen DNAeasy Blood and Tissue kit (Qiagen). DNA samples from both the skin and WB were eventually shipped on dry ice to the Laboratory Reference and Research Branch, Division of STD Prevention, at the CDC in Atlanta, Georgia for diagnostic PCR testing. Samples were tested using a real-time PCR targeting the *Tp polA* gene (Gene Bank Accession No. U57757) as described below.

PCR amplification was performed using forward primer TP-1 (5′CAGGATCCGGCATATGTCC3′), reverse primer TP-2 (5′AAGTGTGAGCGTCTCATCATTCC3′), and probe TP-3 (5′CTGTCATGCACCA GCTTCGACGTCTT3′) as previously published [24] with some exceptions. The probe was labeled with Cyanine (Cy5) at the 5′ end and black-hole quencher 3 (BHQ3) at the 3′ end. PCR was performed in 50µl reaction volumes containing the following: 4µl of deoxynucleoside triphosphate mix (2.5mM of dATP, dCTP, dGTP, and 5.0mM of dUTP), 6µl of MgCl_2_ (25 mM), 0.2µM of each primer, 0.6U uracil N-glycosylase, 5U of AmpliTaq Gold polymerase, 5µl of 10× PCR buffer (All Applied Biosystems, Foster City, CA), 0.2µM of probe, and 12µl of template DNA. Thermocycling was performed in a Rotor-Gene 6000 instrument (Qiagen) as follows: 50°C for 2 min and 95°C for 10 min and 45 cycles of 95°C for 20 sec and 60°C for 1 min. Each PCR run included positive and negative (no template) control reactions. The *Tp* copy numbers for each WB specimen were extrapolated from the standard curve generated using ten-fold serial dilutions of purified *Tp* DNA. A human ribonuclease (RNase) P gene PCR assay was used, as previously described [25], to test for PCR inhibition in blood samples that were negative by the *polA* real-time PCR assay.

### *T. pallidum* Molecular Typing

Strain typing was attempted for all WB and skin samples obtained from SS patients, which were *Tp*-positive by diagnostic PCR. PCR amplification and sizing of the 60-bp tandem repeats within the *arp* (acidic repeat protein) gene and PCR-restriction length polymorphism (RFLP) analysis of *tpr* (*T. pallidum* repeat) *E*, *G*, and *J* genes was done as previously described by Pillay et al. [26], [27], with two modifications. First, the tandem repeat region within the *arp* gene was amplified with a new primer pair, N1 (5′ATCTTTGCCGTCCCGTGTGC3′) and N2 (5′CCGAGTGGGATGGCTGCTTC3′) using the existing PCR conditions for the *arp* assay. Second, all PCR amplicons were analyzed on an Agilent 2100 Bioanalyzer (Agilent Technologies, San Diego, California).

## Results

### Socio-Demographic Features of Secondary Syphilis Patients

A total of 57 patients (age 18–64 years, median 31 years) met the case definition for SS and were recruited by a network of primary health care providers located throughout Cali (Figure 1). All enrolled SS patients resided in comunas of the city of low or very–low socio-economic conditions. Most participants were either unschooled (10.5%) or had only partially completed elementary school education (59.6%). Because we did not directly target more affluent communities in Cali, we are unable to decisively conclude that SS principally affects underprivileged groups. Consistent with prior SS case series [14], the majority of SS patients enrolled were women (64.9%). Most participants were either Afro-Colombian (38.6%) or of mixed race (mestizo) (56.1%). Given that 25.3% of the general population in this region of the country is of Afro-Colombian background [28], it is evident that in this study blacks were proportionately over-represented. The majority of subjects self-reported a high-risk sexual behavior history (62.5%), including having multiple sexual partners, rarely using condoms, and/or having contact with commercial sex workers. Almost a quarter (24.6%) gave a history of illicit drug use and 59.6% stated that they consumed alcohol routinely. One male patient admitted to having had unprotected sex with other men as the principal risk factor for acquiring syphilis. 5.3% of the subjects enrolled in our study were newly diagnosed with HIV. A diagnosis of HIV co-infection is consistent with available epidemiologic and biologic evidence demonstrating that infection with *Tp* increases the likelihood of both transmitting and acquiring HIV [12], [29]–[31].

### Secondary Syphilis Patients Clinical and Laboratory Findings

**Figure 2:**
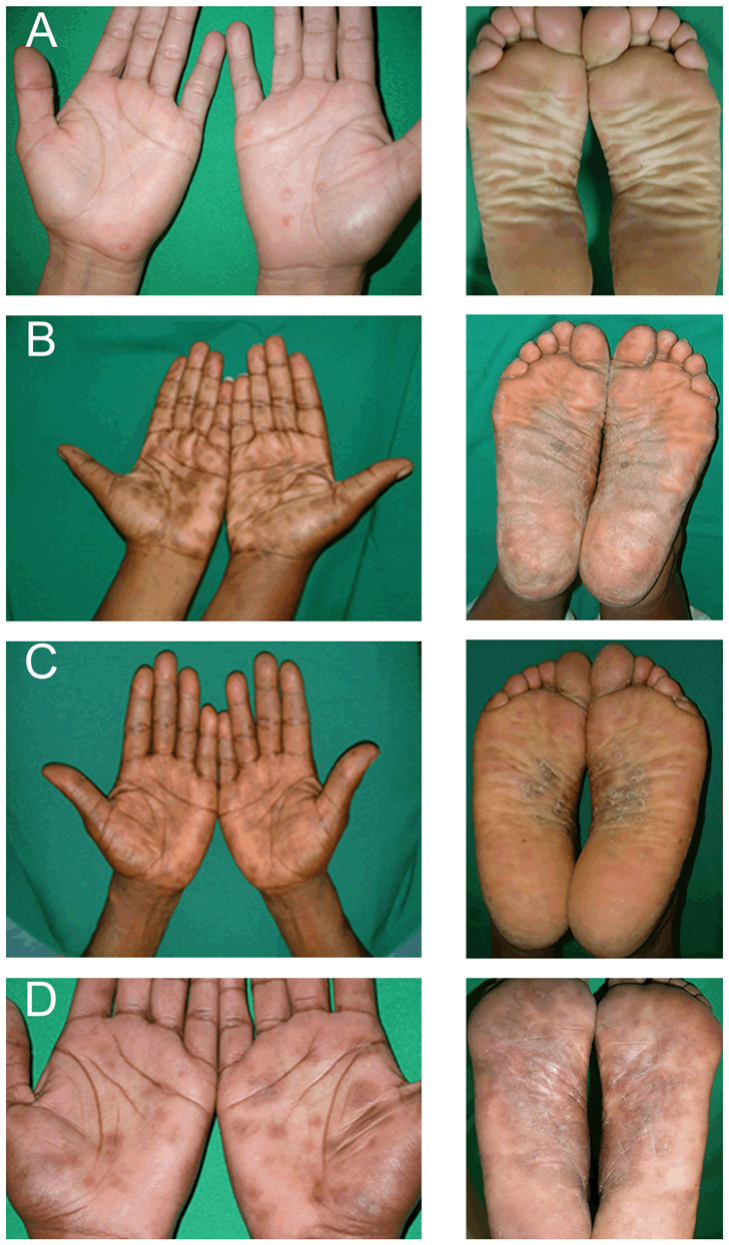
Palmar and plantar rash of secondary syphilis. Typical palmar and plantar rash of secondary syphilis is shown in the representative figures. Similar lesions were evident in 59.6% of all secondary syphilis subjects enrolled. These lesions consist of smooth or scaly plaques and papules, which can become hyperpigmented in dark-skinned individuals as shown in the figure.

**Figure 3:**
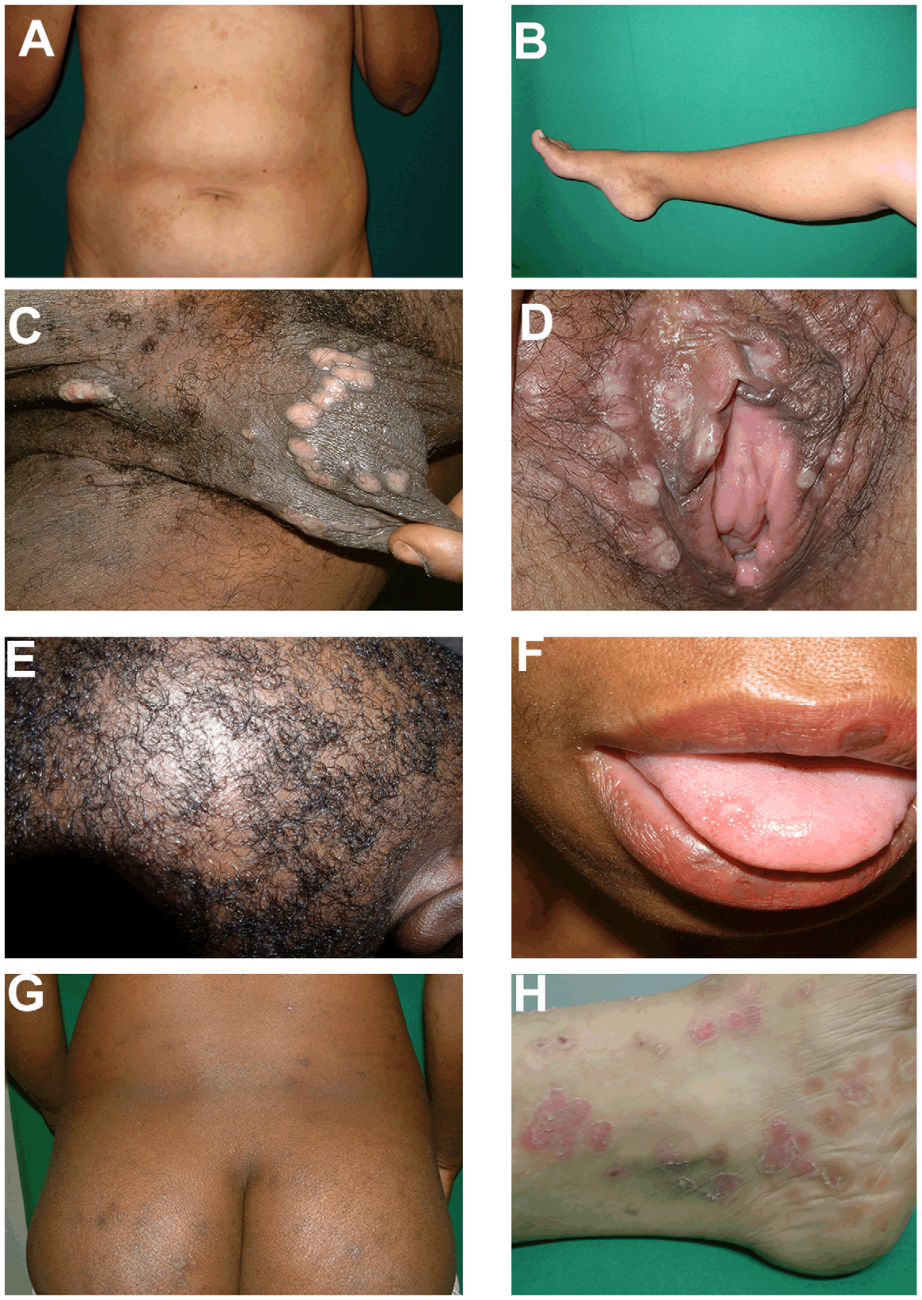
Mucosal and cutaneous lesions of secondary syphilis. Secondary syphilis has been known as the “Great Imitator” due to the diversity of dermatologic lesions and which can be confounded with other cutaneous diseases. (A and B) Diffuse erythematous papular exanthem is shown over abdomen in A and lower extremity in B. (C and D) Multiple moist, hypopigmented, flattened plaques consistent with condyloma lata are shown on external genital areas (male and female respectively in C and D). (E) Inflammatory responses can affect hair follicles leading to “moth-eaten alopecia” as depicted in the figure. (F) Oral mucosal patches, as shown in the figure can be present during secondary syphilis. (G) Pigmentary plaques are shown over the buttocks of a dark-skinned secondary syphilis patient. (H) Psoriasiform syphilitic lesions, as shown in the representative micrograph, could easily be misdiagnosed as psoriasis.

The mean duration of signs and symptoms at the time of presentation was 78.6 days (range 5–240), with a median duration of 30 days. A history of undocumented fever was not uncommon (15.8%); however, none of the patients enrolled was febrile at the time of the initial physical examination. Many patients reported mild to moderate flu-like symptoms (42.1%), and all had dermal and mucosal findings which were in accord with previously described dermatologic manifestations of SS [32], [33] (Table 1). The typical palmar and plantar exanthem of SS [13], [15], [19], [32]–[34] was the most common dermal manifestation (59.6%). As seen in figure 2, palmar and plantar lesions were often surrounded by hyperkeratosis and thin white rings or collar of scales; the latter has been classically known as Biett's collarette [34]. In agreement with Chapel's [32] and Mindel's classic descriptions of SS [32], [33], most subjects also had faint macular and papular eruptions, which were diffusely disseminated over the trunk and upper and lower extremities (Figure 3A–B). In many cases, the dermal lesions were hyperpigmented (Figure 3G), a finding which, not surprisingly, was more evident in darker skinned individuals. Condyloma lata, which are known to be highly infectious [1], [13], [14], were present in 15% of patients (Figure 3C and D). In one subject, several very large frambesiform, pustular lesions, a rare manifestation of the disease [34], were plainly evident over the naso-labial folds and lower jaw (not shown). Three patients had the characteristic patchy “moth-eaten-like” alopecia (Figure 3E), which resolved after penicillin treatment. Mucosal patches in genital areas and/or the oral mucosa, also commonly seen in this stage of the disease, were observed in 14% of patients (Figure 3F). Although early dissemination to the central nervous system (CNS) is known to occur in up to 40% of SS patients [1], [35], [36], none of the participants had overt clinical manifestations associated with meningitis or encephalitis (i.e. meningismus, cranial nerve disorders, visual changes or intense headache). The majority of patients (94.7%) had RPR titers ≥than 1∶16 and over a third (36.8%) had titers ≥1∶128. RPR titers at follow-up decreased at least four-fold in all patients followed at 40–60 days. Hematologic anomalies, indicative of the systemic inflammatory nature of this stage of the disease, were the norm in most patients studied. Indeed, more than half of all patients had an elevated erythrocyte sedimentation rate (ESR) (63.2%), 30% were anemic and several were lymphopenic (43.9%). It is possible, although unlikely, that the hematologic changes described herein are a reflection of other conditions which might be present in underserved individuals from Cali.

**Table 1:** Signs and symptoms associated with secondary syphilis.

Symptom and Signs	No. (%) of Patients		
	This Study	Chapel *	Mindel et al. **
Headache	8/57 (14)	9/105 (9)	17/854 (2)
Fever	9/57 (16)	5/105 (5)	45/822 (6)
Malaise	14/57 (25)	No data	106/831 (13)
Weight loss	3/57 (5)	2/105 (2)	No data
Musculoskeletal aches	3/57 (5)	6/105 (6)	No data
Pruritus	8/57 (14)	44/105 (42)	No data
Adenopathy	14/57 (25)	90/105 (86)	490/780 (63)
**Type of skin lesions**			
Diffuse exanthem	31/57 (54)	7/105 (7)	775/830 (93)
Macular	9/57 (16)	10/105 (10)	301/763 (40)
Maculopapular	9/57 (16)	73/105 (70)	301/763 (40)
Papular	7/57 (12)	13/105 (12)	78/763 (10)
Papulo-pustular	1/57 (2)	2/105 (2)	No data
Psoriasiform	1/57 (2)	1/105 (1)	No data
Condylomata lata	7/57 (12)	9/105 (9)	37/821 (5)
Hair loss	3/57 (5)	3/105 (3)	32/824 (4)
**Site**			
Palms	30/57 (53)	54/105 (55)	346/775 (45)
Soles	32/57 (56)	62/105 (59)	337/773 (44)
Trunk	15/57 (26)	61/105 (58)	571/788 (73)
Genitals	13/57 (23)	58/105 (55)	265/759 (35)
Mucosa	15/57 (26)	22/105 (21)	No data

*[32].

**[33].

### Histological Findings

**Figure 4:**
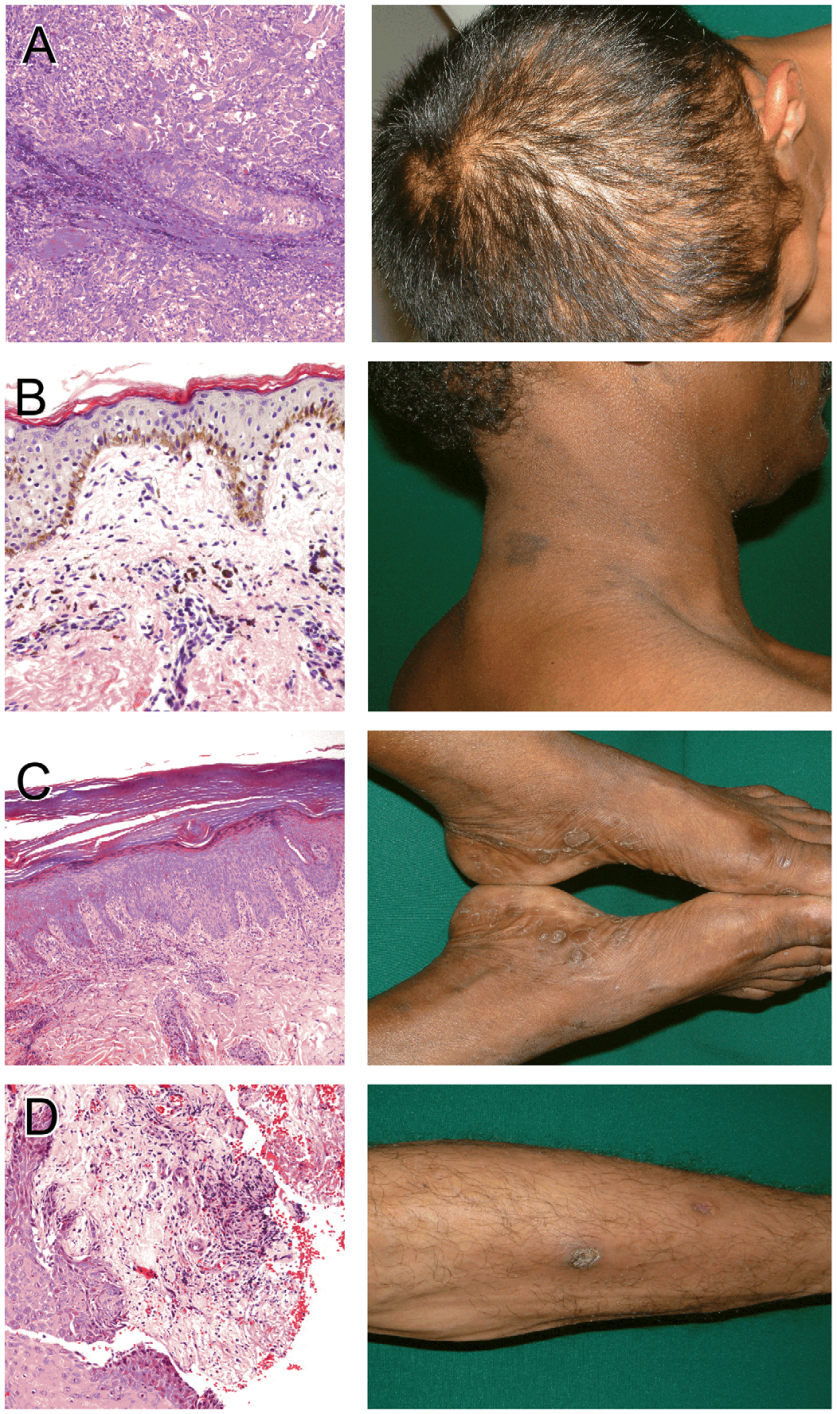
Secondary syphilis histopathology. The figure shows histopathologic anomalies seen in punch biopsies obtained from four secondary syphilis patients skin lesions. Corresponding clinical appearance of the lesions are also shown. (A) Markedly inflamed hair follicle (“folliculitis”) with extension of inflammatory cell infiltrate into parafollicular blood vessel and connective tissue. Corresponding “moth-eaten” alopecia is shown in the adjacent micrograph. (B) Dark-skinned patient (pigmented basal keratinocytes); further darkening of a patch of skin in the form of a macule, as shown herein, is caused by deposition of dermal melanophages (“pigment incontinence”) (C) Skin biopsy obtained near the sole reveals a thick stratum corneum layer, epidermal reactive psoriasiform hyperplasia associated with chronic inflammation of the dermal papilla, and a superficial perivascular lymphoplasmocytic infiltrate. (D) Edge of an ulcer located in the lower extremity reveals fibrinoid exudate on the ulcer bed, surrounded by granulation tissue and reactive hyperplasia of the epidermis.

Table 2 summarizes the various histopathologic anomalies that were seen in lesional punch biopsies obtained from 11 of the 57 SS patients enrolled. In concert with prior histologic descriptions of SS [14], [34], [35], [37]–[40], skin biopsies revealed superficial and deep dermal cellular infiltrates of varying intensity, often in a perivascular distribution and primarily comprised of lymphocytes and plasma cells. Several other histologic patterns involving the epidermis were also seen. These included areas of focal spongiosis, basal vacuolar changes, parakeratosis, and epidermal acanthosis. Figure 4 depicts representative histopathologic abnormalities from four SS patients. In concert with the known low sensitivity of the Warthin-Starry stains to detect *Tp* in tissues [39], [40], spirochetes were only seen in one (9%) of the 11 biopsies studied.

**Table 2:** Histologic abnormalities observed in secondary syphilis skin lesions (n = 11).

Characteristic Features of Inflammatory Infiltrate	N (%)
**Degree of inflammation**	
Sparse	5 (45)
Moderate	2 (18)
Dense	4 (36)
**Location**	
Superficial	10 (91)
Deep	2 (18)
Perivascular	9 (82)
Diffuse	2 (18)
Periadnexal	4 (36)
**Predominant inflammatory cells**	
Lymphocytes	++++
Plasmocytes	+++
Macrophages	++
Neutrophils	+
Eosinophils	+
**Epidermal changes**	
Exocytosis	11 (100)
Necrotic keratinocytes	4 (36)
Spongiosis	11 (100)
Psoriasiform alterations	5 (45)
Acanthosis	8 (73)

### Detection of *Tp* DNA (*pol*A) in Whole Blood (WB) of Secondary Syphilis Patients by Real-Time Quantitative PCR (qPCR)

We previously determined that WB was the best sample to detect spirochetes in blood obtained from infected rabbits [41]. In unpublished observations we confirmed that using spiked human blood, WB was better than serum, plasma or peripheral blood mononuclear cells (PBMCs) for detecting spirochetes. In this study, we therefore elected to use WB samples to quantitate the level of spirochetemia in untreated SS patients. Using a real-time qPCR assay targeting the *polA* gene, we first established the sensitivity of the assay to be between 15 and 150 spirochetes/ml, irrespective of whether freshly extracted *Tp* DNA or live whole spirochetes were used to spike the blood sample (Table S1). The *pol*A PCR detection limit in blood samples that were immediately refrigerated upon spiking and kept at 4°C for a total of 26 hrs prior to DNA extraction, were one log higher than samples kept at room temperature for 1 hr and then processed. We then used this highly sensitive PCR method to amplify *Tp* DNA in 46% (11/26) of the WB samples obtained from SS patients (Table 3). The *polA* copy numbers in these patients ranged from 194.92 to 1954.2 copies/ml of WB, which is well above the cutoff for the assay. PCR inhibition was not observed in any of the DNA samples that tested negative for the *Tp polA* target. It is important to note that the ability to detect *Tp* DNA in SS patients' samples greatly improved when WB DNA was extracted within a few hours of procurement of the sample. Indeed, 63% (7/11) of the samples handled in this fashion had detectable *Tp* DNA, whereas only 5/15 (30%) WB samples were positive when WB was collected, frozen and shipped to UCHC for subsequent DNA extraction at the CDC. These findings highlight the importance of timely specimen processing and handling, and show how subtle differences in technique can greatly alter the ability to amplify *Tp* DNA.

**Table 3:** Quantitation of *T. pallidum* DNA in whole blood (WB) samples by real-time PCR (n = 26).

Subject #	Sample	*polA* diagnostic PCR	Ct value	Copies/mL blood **	RPR titer
20	WB	−	−	−	1∶4
27	WB	+	35.65	482.0	1∶128
28	WB	−	−	−	1∶64
29	WB	−	−	−	1∶128
34	WB	−	−	−	1∶16
39	WB	+	36.24	322.4	1∶16
40	WB	+	35.96	392.3	1∶32
42	WB	+	34.84	847.2	1∶256
43	WB	+	36.32	304.9	1∶64
44	WB	−	−	−	1∶16
50	WB	−	−	−	1∶64
56	WB	−	−	−	1∶16
57	WB	−	−	−	1∶128
58	WB	−	−	−	1∶256
59	WB	−	−	−	1∶16
*64	WB	+	35.67	479.8	1∶256
*71	WB	+	36.15	342.4	>1∶256
*72	WB	+	34.82	857.2	1∶64
*73	WB	+	34.62	989.6	1∶128
*74	WB	+	36.97	194.9	1∶128
*76	WB	+	33.63	1954.2	1∶64
*77	WB	−	−	−	1∶128
*80	WB	−	−	−	1∶256
*84	WB	+	35.1	612.3	1∶128
*87	WB	−	−	−	1∶256
*96	WB	−	−	−	1∶64
		% positive: 46%			

*DNA was extracted immediately after obtaining the samples.

**Reliable detection limit of the assay was between 15 and 150 copies/ml.

### *Tp* Was Readily Detected in Skin Samples of SS Patients

*Tp* DNA was also detected by qualitative real-time PCR in 8/12 (66%) skin biopsies studied (Table 4). Three of the four skin biopsies that did not have detectable *Tp* DNA by RT-PCR were obtained from hyperkeratotic plantar plaques. Five of the 8 patients had *Tp* DNA present in both the blood and the skin, one patient with spirochetemia had a negative PCR in the skin; and two patients with detectable DNA in punch biopsy material had negative PCR results in WB.

**Table 4:** Patients and strain type analysis using DNA obtained from skin lesions.

Subject	WB *pol*A PCR	Skin *pol*A PCR	Strain type (Skin)	Punch biopsy site	Description of the lesion
71	+	+	22a	Epigastrium	Erythematous Plaque
72	+	+	14d	Posterio lumbar	Erythematous Plaque
73	+	+	13d	Right iliac crest	Erythematous Plaque
74	+	−	−	Right sole	Plaque
75	NA	−	−	Left sole	Plaque
76	+	+	16d	Posterior neck	Lichenoid plaque
77	−	+	16d	Right ankle (external maleolus)	Psoriasiform Plaque
80	−	−	−	Right sole	Plaque
84	+	+	14d	Posterior neck	Lichenoid plaque
87	−	−	−	Right leg (distal tibia)	Plaque
96	−	+	−	Posterior lumbar	Hyperpigmented plaque
99	NA	+	?a	Left anterior thigh	Erythematous Plaque

### *Tp* Strain Typing

None of the WB samples analyzed had sufficient quantity of *Tp* DNA to satisfactorily perform molecular strain typing. On the other hand, six of the eight positive skin samples were amenable for typing. By combining the 60-bp *arp* repeat sizes and the *tpr E*, *G*, and *J* RFLP patterns, we identified four different strain types; two each were 14*d* and 16*d*, and one each was 13*d* and 22*a*, respectively. The remaining strain was partially typeable by *tpr* RFLP analysis (pattern *a*). Although it is not possible to generalize our results due to the small sample size, our data suggests high strain diversity, which reflects the pattern seen in South Africa [27], where syphilis is known to be highly endemic.

## Discussion

The WHO estimates that up to a quarter of all yearly cases of infectious syphilis occur in Latin America and the Caribbean [3]. Because syphilis is not rigorously notified, and often not recognized by health care providers in the region, country-specific disease prevalence and incidence rates most likely underestimate the true magnitude of the problem. Existing published studies do provide evidence that venereal syphilis is not only highly endemic but also a very important sexually transmitted disease in tropical regions of the Americas [42]–[57]. For instance, syphilis has been shown to be a leading cause of genital ulcerative disease in both Peru and the Dominican Republic [56], second only to genital herpes. Likewise, in another study from Peru, the prevalence of venereal syphilis was estimated to be 10.5% amongst MSM and 2.0% in socially marginalized men and women [42]. In a Brazilian study, high syphilis prevalence rates were the norm in prisoners, commercial sex workers, and MSM [47]. High syphilis prevalence rates have also been documented for MSM (5% and 13%) and female sex workers (6.8% and 15.3%) in Honduras and Guatemala respectively (Source: PAHO web site http://new.paho.org accessed July, 2009). In one of the few available studies describing the epidemiology of venereal syphilis in Colombia, 10% of female sex workers in Bogota had serologic and clinical evidence of the disease [51]. Although the current study was not designed to be a comprehensive epidemiologic or microbiologic investigation of syphilis in Cali, our combined observations do provide further evidence that venereal syphilis is highly endemic in this region of Colombia. This assertion was further substantiated by the very high syphilis seroprevalence rates documented in 15–24 year old sexually active men and women from poor Cali districts [7].

Our findings also make evident that early syphilis patients in Cali may go undiagnosed and untreated for several weeks, in some cases for several months. This is not at all surprising given that the clinical manifestations of SS are often subtle and can be easily overlooked and/or dismissed by primary care providers and patients alike. A principal objective of the current case series is thus, to review for health care providers, particularly those who practice medicine in tropical regions like Colombia, the typical clinical manifestations associated with SS. Although a large proportion of patients presented with the classic palmo-plantar exanthem of SS [19], [32], [34], some had faint hypo or hyper-pigmented macules and papules that could have easily been dismissed for other dermatologic conditions. Indeed, the rash of SS may be frequently confused for other skin disorders including pityriasis rosea, psoriasis, seborrheic dermatitis and dermato-mycosis, amongst others [13]. Given the relative high prevalence of venereal syphilis in this population, it is not unreasonable to suggest that a diagnosis of SS should be considered in all sexually active individuals who present with any form of unexplained skin and mucous membrane pathology, and perhaps even in individuals with otherwise unexplained constitutional flu-like symptoms.

Several other investigators have previously used molecular methods to detect *Tp* DNA in WB and tissues from untreated early syphilis patients [58]–[65]. Our study is the first to measure spirochetal loads by real time qPCR in the blood of untreated SS patients. The ability to detect spirochetal DNA in these samples has been quite variable and highly dependent on various factors including; the type of sample collected, the stage of the disease (primary vs. secondary vs. latent), and the gene target used. Nevertheless, it is readily apparent that a significant proportion of early syphilis patients, regardless of the stage of the disease, have circulating spirochetal DNA. In the current study, the detection limit in blood samples that were spiked with either DNA or whole *Tp* organisms and kept at 4°C for 26 hrs was 15 *polA* copies/ml blood compared to 150 *polA* copies/ml in the same dilutions stored at room temperature for 1 hr. While frozen blood samples, stored over several days, may not be conducive to diagnostic PCR testing, our spiking experiments do indicate that blood samples can be stored up to 26 hours at 4°C without significantly affecting the capacity to detect *Tp* DNA. This may prove to be particularly useful in settings where blood samples cannot be processed on the day of collection. No PCR inhibition was observed with the use of 2ml blood for spiking experiments, despite the inherent high concentration of human DNA in these samples. It is possible that several other SS patients enrolled, if not all, were spirochetemic but not detected by qPCR. This may have been due to some patients having spirochetemia which was below the threshold of the assay or alternatively, treponemal DNA might have been degraded as a result of not being extracted within 24 hours of blood collection. For future studies the ability to detect low copy numbers may be enhanced by extracting DNA on site and using a larger volume of blood (2ml vs. 400µl).

In this study we also performed *Tp* strain type analysis in DNA material obtained from the skin of several SS patients. Using the method described by Pillay [27], [66], [67] we provide evidence that in the city of Cali there is considerable heterogeneity in circulating Tp strains. Subtype 14d, which is known to have a worldwide distribution [27], [62], [68], was present in the skin of two SS patients. Subtypes 13d and 16d, which were previously identified in syphilis patients in several cities in South Africa [27], were also present in skin samples in Cali. Strain type diversity in Cali, provides additional evidence that venereal syphilis is highly endemic in this population. In a much larger molecular epidemiology study conducted in several South African cities [27], greater geographic *Tp* strain diversity was found to correlate statistically with higher syphilis prevalence rates. It is our contention that an improved understanding of the heterogeneity of syphilis subtypes, not only helps to identify the introduction of new strains into an endemic population but also helps to evaluate if public health strategies have been successful at eradicated indigenous strains.

As already alluded to above, the molecular methods used herein provide evidence that significant numbers of spirochetes are not only present in the skin, but are also capable of spreading in significant numbers through the blood stream of untreated SS patients. Paradoxically, SS patients exhibit robust cellular and humoral adaptive immune responses [10], [17], [69], [70]. A careful analysis of *Tp's* unique ultrastructural features provides several explanations for this paradox. Unlike the outer membrane of gram-negative bacteria, that of *Tp* lacks the potent proinflammatory glycolipid lipopolysaccharide (LPS) [71], [72]. In addition, freeze-fracture microscopy studies have shown that the spirochete is largely devoid of integral outer-membrane proteins [73], [74]. Although *Tp* does contain an abundance of highly antigenic hydrophilic polypeptides, these molecules are tethered by covalently bound N-terminal lipids to the periplasmic leaflet of the cytoplasmic membrane [74], [75]. This unusual topology is thought to contribute to the ability of intact spirochetes to avoid recognition by innate immune cell (i.e. macrophages) pattern recognition receptors (PRR); thus delaying or impeding their activation at the sites of initial inoculation or in skin or organs to where spirochetes have disseminated. Inefficient antibody binding to the small number of potential antigenic targets present on the spirochete's outer membrane could also allow the spirochete to shun rapid and efficient binding by opsonizing anti-treponemal antibodies and thus avoid phagocytosis. One can envision a model were the paucity of outer membrane antigenic targets, perhaps in combination with the very slow rate of bacterial replication, facilitates intermittent low level spread of the spirochete from affected skin and mucous lesions into the blood stream of untreated SS patients. Lastly, sequence variation of the *Tp* repeat (Tpr) family of polymorphic multi-copy repeat proteins has been postulated as an additional mechanism of immune evasion and persistent infection by the spirochete [17], [76]–[78]. Of the several proteins with predicted outer membrane location, TprK has received the most attention. Although controversy remains as to the actual location of TprK [79], sequence diversity of *tprK* in samples obtained from several syphilis patients [76] has bolstered the idea that this molecule could play an important role in immune evasion. Several other candidate outer membrane proteins, including Tp92 [80], are currently being studied to determine if they meet the structural features of other known bacterial outer membrane proteins, if they bind syphilitic opsonic antibodies, and if they too can undergo antigenic variation.

We conclude that high syphilis prevalence rates in the region should prompt health care workers in countries like Colombia to maintain a high index of suspicion for the common and uncommon manifestations of early syphilis. In concert with the clinical findings highlighted herein, a diagnosis of SS must be considered as part of the differential diagnosis in any subject who presents with chronic skin and/or mucosal lesions. Public health care authorities must redouble their efforts to enhance early detection of venereal syphilis, to institute timely treatment of the disease and to improve follow-up of patients diagnosed with the disease. Lastly, research efforts designed to better understand the immunopathogenesis of the disease, in particular how the bacterium is able to elude host immunologic defenses and spread from sites of bacterial replication, as demonstrated herein, will greatly contribute to more effective and novel prevention strategies, including the development of an effective vaccine.

## Supporting Information

Table S1 Sensitivity of polA real-time PCR.
(0.03 MB DOC)
