# Supplementary material for: Secondary Syphilis in Cali, Colombia: New Concepts in Disease Pathogenesis
Source: PLoS Negl Trop Dis. 2010 May 18;4(5):e690. doi: 10.1371/journal.pntd.0000690 (PMC2872645; doi:10.1371/journal.pntd.0000690)
Supplement: Table S1 — Sensitivity of polA real-time PCR. (0.03 MB DOC) [file pntd.0000690.s001.doc]

**Supplemental Table 1. Sensitivity of *pol*A real-time PCR**

| **Spiked sample** | **Incubation Time** | **Sensitivity** |
| --- | --- | --- |
| Extracted *T. pallidum* DNA in PBS | 1hr at room temperature | 15 – 150** spirochetes/mL |
| Fresh live *T. pallidum** in whole blood | 1hr at room temperature | 150 spirochetes/mL |
| Fresh live *T. pallidum** in whole blood | 26 hrs at 4^o^C | 15 spirochetes/mL |
| Purified DNA in blood | 26 hrs at 4^o^C | 15 copies of Tp *pol*A/mL |
| Purified DNA in blood | 1hr at room temperature | 15-150** copies of Tp *pol*A/mL |

*DNA extracted from whole *T. pallidum* organisms spiked in blood.

**One of the duplicate samples tested positive at 10^5^ dilutions
